# Evaluation of the EUROIMMUN automated chemiluminescence immunoassays for measurement of four core biomarkers for Alzheimer’s disease in cerebrospinal fluid

**DOI:** 10.1016/j.plabm.2024.e00425

**Published:** 2024-09-05

**Authors:** Katharina Römpler, Philipp Arendt, Britta Brix, Viola Borchardt-Lohölter, Anette Schulz, Mandy Busse, Stefan Busse

**Affiliations:** a Institute for Experimental Immunology, Affiliated to EUROIMMUN Medizinische Labordiagnostika AG, Seekamp 31, 23560, Luebeck, Germany; b Department for Experimental Obstetrics and Gynecology, Otto von Guericke University Magdeburg, Medical Faculty, Gerhart-Hauptmann-Str. 35, 39180, Magdeburg, Germany; c University Hospital for Psychiatry and Psychotherapy, Otto von Guericke University Magdeburg, Leipziger Str. 44, 39120, Magdeburg, Germany

**Keywords:** Alzheimer's disease, Biomarkers, Cerebrospinal fluid, Chemiluminescence, Immunoassay, Validation

## Abstract

**Introduction:**

Robust immunoassays for quantification of Alzheimer's disease (AD)-specific biomarkers are required for routine diagnostics. We report analytical performance characteristics of four new chemiluminescence immunoassays (ChLIA, EUROIMMUN) running on closed, fully automated random-access instruments for quantification of Aβ_1-40_, Aβ_1-42_, tTau, and pTau(181) in human cerebrospinal fluid (CSF).

**Methods:**

ChLIAs were validated according to the guidelines of the Clinical and Laboratory Standards Institute (CLSI). Optimal cut-offs for biomarkers and biomarker ratios were determined using samples from 219 AD patients and 220 patients with AD-related symptoms. For performance comparison, biomarker concentrations were measured in 110 diagnostic leftover samples using the ChLIAs and established Lumipulse G assays (Fujirebio).

**Results:**

All ChLIAs met CLSI criteria. Overall agreement between assays was 89.0%–97.3 % with highly correlating results (Pearson's correlation coefficients: 0.82–0.99). Passing-Bablok regression analysis revealed systematic differences.

**Discussion:**

EUROIMMUN ChLIAs showed good analytical performances and represent new valuable tools for diagnostics of AD.

## 1 Introduction

Alzheimer's disease (AD) is a neurodegenerative disease with progressing cognitive impairment and the main cause of dementia in persons over 65 years of age. The disease is pathophysiologically based on the formation of plaques and neurofibrillary tangles, as well as the degeneration of neurons and synapses [[1], [2], [3]]. The AD-related biomarkers beta-amyloid (1–40) (Aβ_1-40_), beta-amyloid (1–42) (Aβ_1-42_), total Tau (tTau) and Tau phosphorylated at threonine 181 (pTau(181)) have gained great importance [[4], [5], [6]] and are of essential use for laboratory analytics supporting the clinical diagnosis [3,7]. The Aβ_1-42_ concentration in the cerebrospinal fluid (CSF) of AD patients is inversely proportional to the amount of amyloid plaques and is approximately 50 % lower than in cognitively healthy elderly persons [8]. Aβ_1-40_ shows no or only small changes. Importantly, the ratio Aβ_1-42_/Aβ_1-40_ is more reliable than Aβ_1-42_ as a single biomarker because individual differences in the production of amyloids can be neglected and negative pre-analytical effects, caused, e.g., by the material and size of the sample tubes and freeze/thaw cycles can be eliminated [4,9]. tTau is an unspecific marker of neurodegeneration [10] that is increased in CSF of AD patients. CSF pTau(181) is considered a fairly specific biomarker, with significantly increased levels in AD patients [11].

Whether biomarkers can make a meaningful contribution to diagnosis depends on the quality of the results derived. Collaborative research brought solid advancements regarding established standard operating procedures for lumbar puncture [12,13], international guidelines for handling of CSF samples and standardization of pre-analytical factors [14,15]. Furthermore, standardization efforts with certified reference materials (CRM) for CSF Aβ_1-42_ have harmonized results between assays of different manufacturers, thus paving the way for globally uniform reference limits and cut-off values [1,16,17]. ELISAs for CSF biomarker quantification are still established in clinical laboratories due to their ease of use without need for special equipment [18]. However, the utility of results using ELISAs is challenged by the relatively high impact of influencing factors that may lead to great intra-laboratory and inter-laboratory variability [19]. In contrast to that, stable and high-precision measurements of CSF biomarker concentrations are possible using closed and fully automated random-access instruments. These instruments for chemilumincescence immunoassays (ChLIAs) improve analytical precision and consequently the majority of laboratories now benefit from accurate, fast and flexible test throughput [20,21]. Recent evidence supports the usefulness of CSF biomarkers measured by ChLIA on fully automated random-access platforms for AD diagnosis [[22], [23], [24]].

EUROIMMUN has developed four new ChLIAs for quantitation of Aβ_1-40_, Aβ_1-42_, tTau, and pTau(181) in CSF for fully automated processing. This study aimed to evaluate their analytical performance characteristics and to assess agreements between ChLIA results and those of other established assays.

## 2 Methods

### 2.1 Quantification of AD-related biomarkers

Aβ_1-40_, Aβ_1-42_, tTau, and pTau(181) were quantified in CSF samples using the Beta-Amyloid (1–40), the Beta-Amyloid (1–42), the Total-tau, and the pTau(181) ChLIA (all EUROIMMUN Medizinische Labordagnostik AG, Lübeck, Germany) according to the manufacturer's instructions on the automated system IDS-i10 (Immunodiagnostic systems). Capture antibody (Ab)-coated magnetic particles and detection Abs bind to specific epitopes (Supplementary Table 1) of the respective antigens. In the Beta-Amyloid (1–40) and (1–42) ChLIAs, acridinium-labelled extravidin is added to the biotinylated detection Ab in a second reaction step. tTau and pTau(181) ChLIAs, use an acridinium-labelled detection Ab. Subsequently, trigger solutions induce a chemiluminescence reaction. The resulting light signal (relative light units, RLU) is automatically converted in pg/ml.

For performance assessment, results of the EUROIMMUN ChLIAs were compared with those obtained using the Lumipulse G β-Amyloid 1–40, β-Amyloid 1–42, Total Tau and pTau 181 (all Fujirebio Inc., Tokyo, Japan) performed fully automated on the LUMIPULSE G 600II [25] according to the manufacturer's instructions by an independent contract laboratory.

### 2.2 Analytical validation

All ChLIAs were evaluated in accordance with the guidelines of the Clinical and Laboratory Standards Institute (CLSI).

Intra-lot **precision** (CLSI EP05-A3, within-run, between-run, within-day and between-day reproducibility) was analyzed using six CSF leftover samples (80 determinations per sample) in triplicate on 20 days with two runs per day. Inter-lot precision was analyzed using six leftover samples (90 determinations per sample) in triplicate on five days with three different lots.

For determination of **range of detection capability** (limit of blank (LoB), limit of detection (LoD) and limit of quantitation (LoQ), CLSI EP17-A2) sample buffer and four CSF samples with low AD-related biomarker concentration were tested in total 60 times in replicate with three different lots on three different days.

**Linearity** (CLSI EP06-A) was determined applying three different lots using four replicates of sets of serially diluted leftover samples and sample buffer. Observed and expected results were compared, linearly and non-linearly fitted and the mean coefficient of determination (R^2^) was calculated.

**Interference** (CLSI EP07-A3) was studied using four leftover samples spiked with 1 % whole blood and biotin up to a concentration of 10 μg/ml. The recovery rate of the spiked sample compared with the control sample was calculated.

The analysis of **stability** (CLSI EP25-A) of ChLIA test kits was performed in accordance with the international standard DIN EN ISO 23640:2015 in triplicate using four samples covering the total measurement range of the respective ChLIAs including a negative control. **Transport stability** was determined using one lot before and after a transport simulation of a four-day period at 2 °C−33 °C. To investigate **accelerated stability**, samples were analyzed at various days between zero and 12 (Beta-Amyloid (1–40) and Beta-Amyloid (1–42) ChLIA) or zero and 11 days (tTau and pTau(181) ChLIA) using three different lots stored at 37 °C. The **calibration stability** was tested at 12 °C−15 °C at zero, seven, 14, and 28 days with samples, and additionally calibrators and negative control at time point zero using three different lots. **On-board-stability of cartridges** was investigated using three different lots, of which one has passed the transport simulation, at various days between zero and 60 at 12 °C−15 °C. **On-board-** and **in-use-stability** testing for **calibrators** was performed between zero and 6 h (Beta-Amyloid (1–40) and (1–42) ChLIA), zero and 10 h (tTau and pTau(181) ChLIA), and at various times between zero and 60 days, respectively, between 18 °C and 25 °C. **Stability** and recovery rates of positive and negative controls were determined after three **freeze/thaw** cycles. **Real-time stability of cartridges and calibrators** was determined at 2 °C−8 °C at various times between zero and 24 months.

**Trueness** (CLSI EP09-A3) was analyzed for the Beta-Amyloid (1–42) ChLIA using triplicate of three CRM with different concentrations using three different lots. The overall recovery was determined. As there are no CRMs available for Aβ_1-40_, tTau, or pTau(181), trueness testing was only performed for Beta-Amyloid (1–42) ChLIA.

**Cross-reactivity** was investigated for the Beta-Amyloid (1–40) and the Beta-Amyloid (1–42) ChLIA using the eight different beta-amyloid peptides Aβ_1-38_, Aβ_1-39_, Aβ_1-42_ or Aβ_1-40_, Aβ_2-42_, Aβ_3-42_, Aβ_4-42_, Aβ_11-42_, Aβ_1-43_ at a concentration of 10 ng/ml with three different test lots, and for the pTau(181) ChLIA using three analyte-containing samples each spiked with three different concentrations of Tau441. The respective pTau(181) background values were subtracted from the measured values of the samples spiked with Tau441. Dependent on the recovery rate, cross-reactivity was not probable (0 %–5 %), not excludable (5 %–20 %) or probable (>20 %). The cross-reactivity of the Total-Tau ChLIA was not analyzed, because tTau is a heterogenic group of analytes with different isoforms.

### 2.3 Cut-off determination

For the cut-off determination of the EUROIMMUN ChLIAs, the concentrations of Aβ_1-40_, Aβ_1-42_, tTau, and pTau(181) were measured in CSF samples from 439 patients of a geropsychiatric ward (University Hospital for Psychiatry and Psychotherapy, University Magdeburg). Of those, 219 patients (mean age: 80 years (range:59−95); 101 female, 61 male, 57 unknown sex) were clinically diagnosed with AD and 220 (mean age: 72 years (range:34−94); 77 female, 84 male, 59 unknown sex) had other neurological disorders with AD-like symptoms. Patients were diagnosed according to criteria of the Diagnostic and Statistical Manual of Mental Disorders, Fourth Edition (DSM-IV) by psychiatrists, neurologists, and psychologists. The diagnosis was based on results of magnetic resonance imaging of the brain or cerebral computer tomography, CSF analysis, mini-mental state evaluation, Montreal Cognitive Assessment, and CERAD test battery. Measurement of AD-related biomarkers was performed at the University Magdeburg using INNOTEST® immunoassays (Fujirebio). CSF samples were obtained in the morning and collected in 15 ml high clarity polypropylene centrifuge tubes (Falcon®). Demographics and patient diagnosis is summarized in Table 1. The data were evaluated using receiver operating characteristic (ROC) curve analysis. Optimal cut-offs for the determination of Aβ_1-42_, tTau, pTau(181) as well as Aβ_1-42_/Aβ_1-40_, Aβ_1-42_/tTau and Aβ_1-42_/pTau(181) were derived using Youden's index. All calculations were performed using Analyse-it® Validation Edition.

**Table 1:** Demographics and diagnosis of patients.

Diagnosis of patients	Number	Mean age (years, range)	Sex (female/male/unknown)
Alzheimer's disease	219	80 (59–95)	101 / 61 / 57
Frontotemporal dementia	27	79 (54–86)	4 / 13 / 10
Lewy body disease	7	78 (75–84)	0 / 5 / 2
Vascular dementia	59	78 (56–94)	21 / 20 / 18
Depression	71	72 (46–91)	34 / 21 / 16
Alcohol related dementia	4	61 (58–62)	0 / 3 / 1
Parkinsons disease	6	75 (69–79)	1 / 4 / 1
Schizophrenia/Psychosis	23	63 (34–83)	8 / 10 / 5
Creutzfeldt-Jakob disease	1	59	0 / 0 / 1
Neuroborreliosis	3	72 (72–73)	0 / 3 / 0
Herpes-Encephalitis	1	49	1 / 0 / 0
Wernicke's encephalopathy	1	62	1 / 0 / 0
Corticobasal degeneration	1	56	0 / 1 / 0
Mild cognitive impairment	1	59	0 / 1 / 0
Progressive supranuclear palsy	1	66	0 / 0 / 1
Mixed dementias	14	79 (66–93)	7 / 3 / 4

For Lumipulse G assays, cut-offs recommended by the manufacturer were used: 599 pg/ml (Aβ1-42), 404 pg/ml (tTau), 56.5 pg/ml (pTau(181)), 0.069 (Aβ1-42/Aβ1-40), 1.275 (Aβ1-42/tTau), 8.1 (Aβ1-42/pTau(181)).

### 2.4 Method comparison: EUROIMMUN ChLIAs versus Fujirebio Lumipulse G

The performance of the EUROIMMUN ChLIAs (applying cut-offs as calculated beforehand) was assessed in comparison with the respective Lumipulse G assays using leftover CSF samples from 110 patients with unknown diagnosis for the laboratory investigators (mean age 57.2, ranging from 11 to 94 years; 49 female, 60 males, 1 unknown age and sex). CSF samples were collected between February and December 2021. After completion of all serodiagnostic analyses, samples were aliquoted in polypropylene tubes and stored at −20 °C between 2 and 12 months before measurement of AD-related biomarker concentrations using EUROIMMUN ChLIAs. The measurement using the corresponding Lumipulse G assays occurred approximately 3 months later at a contract laboratory. The samples' biomarker concentrations covered the entire measurement range of the EUROIMMUN ChLIAs. Percentage agreement and Cohen's kappa (ĸ) categorized as follows: 0 < κ < 0.2: slight agreement, 0.21 < κ < 0.4: fair agreement, 0.41 < κ < 0.6: moderate agreement, 0.61 < κ < 0.8: substantial agreement, 0.81 < κ < 1: almost perfect agreement [26] were calculated. For quantitative method comparisons, agreement between biomarker concentrations was analyzed by Passing-Bablok regression and Kolmogorov-Smirnov CUSUM test for linearity at a significance level of 5 %.

### 2.5 Ethics statement

The study was performed in agreement with the Declaration of Helsinki and approved by a local ethics committee (University of Magdeburg, registration number: 134/13 and 21/14). Diagnostic left-over samples were collected by a routine diagnostic laboratory (Lübeck, Germany). All samples were processed anonymously. Informed consent was obtained from subjects involved in this study.

## 3 Results

### 3.1 Analytical validation

#### 3.1.1 Precision

All ChLIAs met the criteria of the CLSI guidelines for precision. The coefficients of variation (CV) for the overall intra-lot precision of the Beta-Amyloid (1–40), the Beta-Amyloid (1–42), the Total-Tau and the pTau(181) ChLIAs were 2.4 %–8.0 %, 2.1 %–4.7 %, 1.9 %–3.3 % and 2.5 %–5.0 %, respectively (Supplementary Table 2). CVs for within-run repeatability were 1.4–6.8 %, 1.8 %–4.2 %, 1.0 %–2.0 %, and 1.3 %–2.6 % and for between-run 0.2 %–2.3 %, 0 %–1.6 %, 0.4 %–1.7 % and 0.3 %–3.4 %, respectively. Within-day and between-day reproducibility testing resulted in CVs from 2.2 % to 6.9 % and 0 %–4.0 %, 1.8 %–4.2 % and 0.7 %–2.1 %, 1.5 %–2.6 % and 1.1 %–2.0 %, and 1.4 %–4.3 % and 2.1 %–2.8 %, respectively.

CVs for overall inter-lot precision (Supplementary Table 3) were between 3.6 % and 14.0 %, 4.8 % and 6.0 %, 3.6 % and 5.0 %, and 4.9 % and 8.3 % correspondingly for the Beta-Amyloid (1–40), Beta Amyloid (1–42), Total-Tau and pTau(181) ChLIAs. The CVs for between-lot reproducibility ranged from 1.7 to 13.1, 3.0−4.6 %, 1.8−3.8 % and from 4.0 to 7.3 %, respectively.

#### 3.1.2 Range of detection capability

LoBs of 15.4 pg/ml, 12.7 pg/ml, 3.9 pg/ml, and 1.8 pg/ml were determined for the Beta-Amyloid (1–40), the Beta-Amyloid (1–42), the Total-Tau as well as the pTau(181) ChLIA, respectively (Supplementary Table 4). LoDs were 28.3 pg/ml, 17.5 pg/ml, 5.6 pg/ml, and 3.1 pg/ml and LoQs 41.0 pg/ml, 45.4 pg/ml, 17.4 pg/ml and 9.2 pg/ml, respectively. The specification LoB < LoD/LoQ < lowest concentration of calibrators or 20 % below cut-off were met for all ChLIAs. The upper LoQ (ULoQ) was defined as 20,000 pg/ml, 2,000 pg/ml, 2,000 pg/ml and 400 pg/ml. Lower LoQ (LLoQ, lowest amount of quantifiable measurand) was determined by plotting the mean LoD values against the respective CVs at the point of intersection at a targeted limit of a CV of 8 %. For the tTau ChLIA, LLoQ was determinable for only one of three lots, since no intersection at a CV of 8 % could be found for the two other lots (Supplementary Fig. 1). Results of LLoQ and ULoQ defined the measurement ranges for the respective ChLIAs.

#### 3.1.3 Linearity

A linear relationship between serially diluted samples was found within the measurement ranges for all ChLIAs with relative nonlinearity less than 15 %. R^2^ for all ChLIAs was >0.99. (Supplementary Fig. 2).

#### 3.1.4 Interference

There was no whole blood (1 %) and biotin (up to 10 μg/ml) interference detectable in any of the ChLIAs (Supplementary Table 5).

#### 3.1.5 Stability

All ChLIAs were stable in the investigated ranges. Data are shown in Supplementary Table 6.

#### 3.1.6 Trueness

CRM for Aβ_1-42_ were recovered between 91.2 % and 110.4 % (Supplementary Table 7) indicating trueness within the CLSI specifications above 88 % and below 112 %.

#### 3.1.7 Cross-reactivity

Using the Beta-Amyloid (1–40) ChLIA, the recovery rate for the different amyloid peptides was <0.5 %, except for Aβ_1-39_ with a recovery rate of 11 %. Hence, cross-reactivity was not excludable for this analyte. Using the Beta-Amyloid (1–42) ChLIA, the different amyloid peptides yielded recovery rates <1 % (Supplementary Table 8). Cross-reactivity of the pTau(181) ChLIA was <0.02 % (Supplementary Table 8).

### 3.2 Cut-off

ROC curves of the AD-related biomarkers (Fig. 1A) and of the amyloid and heterologic ratios were compared (Fig. 1B). The area under the curve (AUC) was 0.78, 95 % CI: [0.74, 0.82] for Aβ_1-42_, 0.91, 95 % CI: [0.88, 0.94] for tTau, and 0.95, 95 % CI: [0.93, 0.97] for pTau(181), 0.92, 95 % CI: [0.89, 0.94] for Aβ_1-42_/Aβ_1-40_, 0.93, 95 % CI: [0.91, 0.96] for Aβ_1-42_/tTau, and 0.94, 95 % CI: [0.92, 0.96] for Aβ_1-42_/pTau(181).

**Fig. 1:**
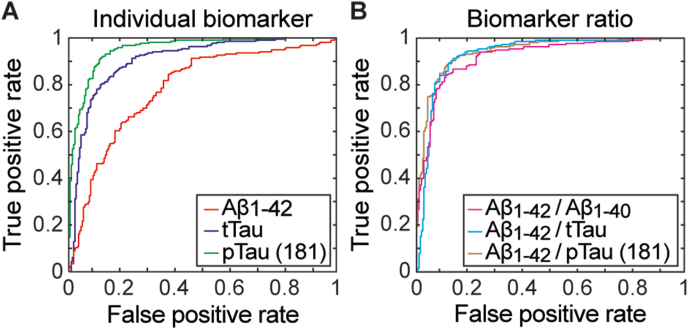
Receiver operating characteristic (ROC) curves for the determination of (A) Aβ_1-42_, tTau, and pTau(181) as well as for (B) the ratios Aβ_1- 42_/Aβ1_-40_, Aβ_1- 42_/tTau, and Aβ_1- 42_/pTau(181) using EUROIMMUN ChLIAs. As the area under the curve (AUC) approaches 1, the test system achieves higher combined values of sensitivity and specificity.

The optimal cut-off was defined using Youden's index. Youden's index for Aβ_1-42_ detection was 0.472, corresponding to an Aβ_1-42_ cut-off of 741 pg/ml (plateaued from 740 to 752 pg/ml, sensitivity: 84.5 %, specificity: 62.7 %, Fig. 2A). For tTau detection, Youden's index was 0.681 relating to a cut-off of 508 pg/ml (plateau: 478–555 pg/ml, sensitivity: 83.6 %, 95 %; specificity: 84.5 %, Fig. 2B). For pTau(181), Youden's index was 0.795 linked to a cut-off of 58.2 pg/ml (plateau: 54.4–58.2 pg/ml, sensitivity: 90.9 %,; specificity: 88.6 %, Fig. 2C) (Supplementary Table 9A). The Youden's index for the ratio Aβ_1-42_/Aβ_1-40_ was 0.731 corresponding to a cut-off of 0.093 (sensitivity: 86.8 %, specificity: 86.4 %, Fig. 2D). The maximum Youden's indices for the heterologic ratios Aβ_1-42_/tTau and Aβ_1-42_/pTau(181) were 0.777 observed at a cut-off of 1.28 (sensitivity: 91.3 %, specificity: 86.4 %, Figs. 2E) and 0.772 at a cut-off of 10.04 (sensitivity: 89.0 %, specificity: 88.2 %, Fig. 2E), respectively (Supplementary Table 9B).

**Fig. 2:**
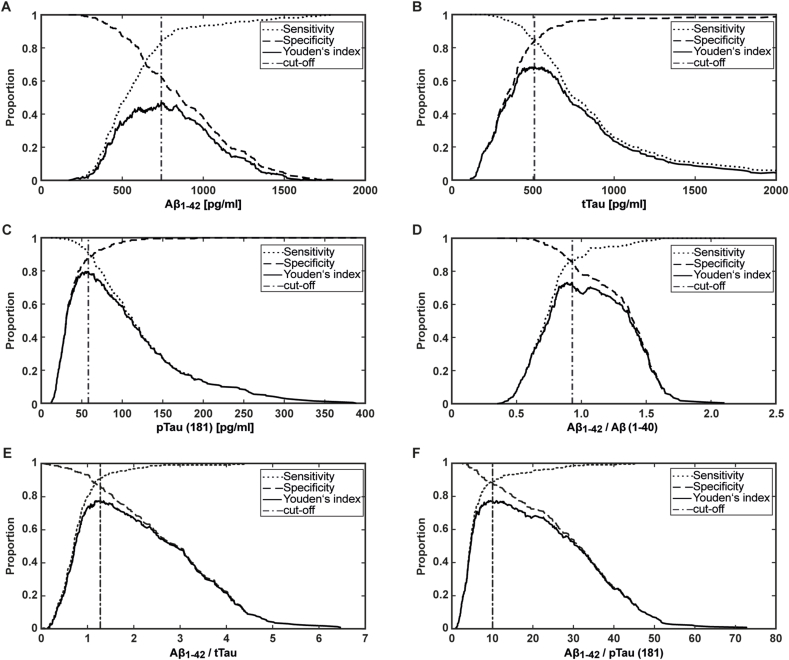
The graphs depict sensitivity, specificity, and Youden's index for the range for the detection of (A) Aβ_1-42_, (B) tTau, (C) pTau(181), (D) Aβ_1-42_/Aβ_1-40_ (E) Aβ_1-42_/tTau, and (F) Aβ_1-42_/pTau(181) using EUROIMMUN ChLIAs. In each subplot, the vertical line marks the cut-off and a plateau range with a fluctuating Youden's index can be observed.

### 3.3 Method comparison: EUROIMMUN ChLIAs versus Fujirebio Lumipulse G

The performance of assays was assessed using 110 CSF leftover samples. The Total-Tau ChLIA detected three samples below LLoQ and one above ULoQ. The Lumipulse G Total Tau found 24 samples below LLoQ. Using the Lumipulse G assays for Aβ_1-40,_ Aβ_1-42_, and tTau determination, the respective concentrations were above ULoQ in one, six and two samples, with one sample, above the ULoQ for Aβ_1-40_ and Aβ_1-42_. This sample was excluded from subsequent considerations. All other samples above ULoQ were evaluated as positive and below LLoQ as negative.

The qualitative method comparison revealed good or almost perfect agreement (ĸ = 0.65 − ĸ = 0.94) with overall agreements between 89.0 % and 97.3 % (Table 2).

**Table 2:** Numbers of samples with normal and abnormal AD-related biomarker concentrations and amyloid and heterologic ratio by means of EUROIMMUN ChLIAs and Lumipulse G assays and results of the qualitative method comparison. N, number of samples; ĸ, Cohen's kappa; CI, 95 % confidence interval.

				Agreement % [CI]			ĸ [CI]
				normal	abnormal	overall	
**Aβ**_**1-42**_		**Fujirebio**		100 [94.0, 100]	94.0 [83.5, 98.8]	97.3 [92.2, 99.4]	0.94 [0.88, 1.0]
	110	normal	abnormal				
**EUROIMMUN**	normal	60	3				
	abnormal	0	47				

**tTau**		**Fujirebio**		93.4 [86.2, 97.5]	79.0 [54.4, 94.0]	90.9 [83.9, 95.6]	0.69 [0.52, 0.87]
	110	normal	abnormal				
**EUROIMMUN**	normal	85	4				
	abnormal	6	15				

**pTau(181)**		**Fujirebio**		93.9 [94.0, 100]	91.7 [83.5, 98.8]	93.6 [92.2, 99.4]	0.72 [0.53, 0.92]
	110	normal	abnormal				
**EUROIMMUN**	normal	92	1				
	abnormal	6	11				

**Aβ**_**1-42**_**/Aβ**_**1-40**_		**Fujirebio**		98.8 [93.5, 100]	57.7 [36.9, 76.7]	89.0 [81.6, 94.2]	0.65 [0.47, 0.83]
	109	normal	abnormal				
**EUROIMMUN**	normal	82	11				
	abnormal	1	15				

**Aβ**_**1-42**_**/tTau**		**Fujirebio**		97.7 [91.9, 99.7]	91.3 [72.0, 98.9]	96.4 [91.0, 99.0]	0.89 [0.79, 1.0]
	110	normal	abnormal				
**EUROIMMUN**	normal	85	2				
	abnormal	2	21				

**Aβ**_**1-42**_**/pTau(181)**		**Fujirebio**		97.9 [92.8, 99.8]	92.3 [64.0, 99.8]	97.3 [92.2, 99.4]	0.87 [0.73, 1.0]
	110	normal	abnormal				
**EUROIMMUN**	normal	95	1				
	abnormal	2	12				

**Fig. 3:**
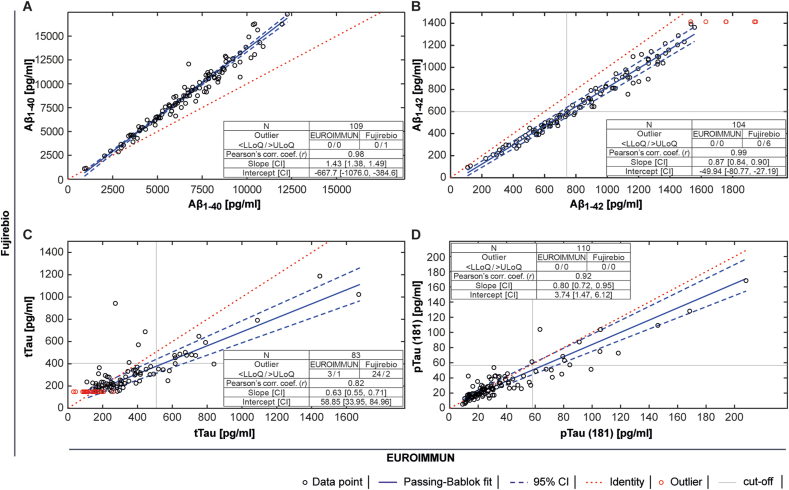
Passing-Bablok regression analysis comparing EUROIMMUN ChLIAs and Lumipulse G immunoassays. (A) Aβ_1-40_, (B) Aβ_1-42_, (C) tTau, (D) pTau(181). The regression line is indicated in solid blue, respective 95 % confidence intervals in dashed blue, and the identity line in dashed red. Slope and intercept denote Passing-Bablok regression parameters for comparisons between the two assays. Values outside the measurement range are plotted as red circles. N, number, LLoQ, lower limit of quantification, ULoQ, upper limit of quantification. (For interpretation of the references to colour in this figure legend, the reader is referred to the Web version of this article.)

The quantitative method comparison revealed constant as well as proportional differences between the assays (Fig. 3). Except for Aβ_1-40_ values, values derived by Lumipulse G assays were generally lower than those derived by the EUROIMMUN ChLIAs (Fig. 3). Aβ_1-42_ assays were highly correlated (Pearson's correlation coefficient *r* = 0.99) and the Passing-Bablok regression showed a slope closest to the identity line (0.87). Lowest correlation (*r* = 0.82) and low slope (0.63) were observed for the tTau assays (Fig. 3). No significant deviation from linearity (p > 0.1) was found for comparisons between immunoassays.

## 4 Discussion

The use of test systems running on fully automated random-access systems for the detection of AD-related biomarkers is highly beneficial for diagnostic laboratories because it improves the analytical precision [20,21]. Here, we show that the EUROIMMUN ChLIAs proved to be specific for their respective target analyte. Interferences were not observed. Measurement of CRM yielded recovery rates similar to those observed by others [17] with repeatable results using several lots. Cross-reactivities were excluded except for the Beta-Amyloid (1–40) ChLIA when spiking a sample with Aβ_1-39_ to a concentration of 10 ng/ml. Aβ_1-39_ is a beta-amyloid isoform that can be found in CSF samples. The *in vivo* concentration of Aβ_1-39_ is approximately 14 % of the concentration of Aβ_1-40_ [27,28], which was found to range between 6.7 and 7.8 ng/ml in CSF samples of different disease cohorts (determined by using Beta-Amyloid (1–40) ChLIA, data not shown). As the concentration of Aβ_1-39_ used in this study to test cross-reactivities greatly exceeds its average *in vivo* concentration, the cross-reaction observed here is irrelevant for laboratory diagnostics. Moreover, since the ratio between Aβ_1-39_ and Aβ_1-40_ seems to be quite stable in different disease conditions [27,28], any misclassification originating from a potential cross-reaction is unlikely as the clinical cut-off applied here for the Beta-Amyloid (1–40) ChLIA was derived using patient samples.

In several studies, cut-offs were derived using samples from AD patients and cognitively healthy individuals. In other studies, control samples for cut-off determination are obtained from patients with neurological diseases without AD-related pathology, such as multiple sclerosis or epilepsy. Presumably, these control patients are of younger age than average AD patients [23,29]. Both approaches bear the risk of misrepresenting conditions of the clinical reality [29] and consequently of misdiagnosing patients. As AD shares symptoms with several neuropsychiatric disorders such as frontotemporal or vascular dementia, using samples from these patients could minimize this risk of misdiagnosis and increase diagnostic accuracy of assays [30]. One great advantage of the present study is that for cut-off determination, samples from patients with similar symptoms and of similar age compared to AD patients were obtained from a geriatric psychiatry. Moreover, a total number of 439 CSF samples was analyzed, which is a higher number of samples compared to those used in various other studies [23,29].

The comparison of results obtained using the EUROIMMUN ChLIAs and the corresponding Lumipulse G assays revealed high overall agreement (89.0 %–97.3 %) as well as good correlation (*r* = 0.82−*r* = 0.99). However, Passing-Bablok regression analysis showed constant as well as proportional differences between test systems. Absolute concentrations differed between tests with results from EUROIMMUN assays showing higher concentrations than Lumipulse G assays, except for Aβ_1-40_ concentrations. A reason for this shift could be the lack of CRM for the analytes Aβ_1-40_, tTau and pTau(181). Assessment of trueness for the Beta-Amyloid (1–42) ChLIA using CRM revealed only minor divergence (0.8 %–10.4 %) to the reference concentrations, which has also been observed for the Lumipulse G β-Amyloid 1–42 assay by others [17]. Nevertheless, Passing-Bablok analysis indicated systematic differences between EUROIMMUN and Lumipulse G assays for the determination of Aβ_1-42_ concentrations. As the Lumipulse G assays were performed by a contract laboratory and EUROIMMUN ChLIAs were run in-house, systematic differences due to inter-laboratory discrepancies cannot be ruled out, although the performance assessment was planned thoroughly to minimize pre-analytical factors. However, these numerical differences have little influence on the diagnostic evaluation since cut-offs of both assays also differ as they were adjusted to samples from different cohorts. Indeed, best agreement was found between Beta-Amyloid (1–42) ChLIA and Lumipulse G β-Amyloid 1–42 (Table 2).

The established Aβ_1-42_/Aβ_1-40_ ratio minimizes the influence of pre-analytical factors and thereby variations between laboratories [31]. This is advantageous if the guidelines for CSF handling and protocols for standardization of pre-analytical factors cannot be met, for instance when CSF samples are sent to centralized diagnostic laboratories that do not implement pre-analytical protocols regarding sample handling or storage, which might affect the measured biomarker concentration [14]. While several initiatives implement algorithms that include most or all of the four individual biomarkers [5,32], some research groups are pushing towards the use of heterologic biomarker ratios [33]. The reason being that the ratio including the two hallmark biomarker of Alzheimer's disease (Aβ_1-42_/pTau(181)) should be sufficient to support the differentiation between Alzheimer's and non-Alzheimer's disease. The heterologic ratios Aβ_1-42_/tTau and Aβ_1-42_/pTau(181) are of increasing interest, because their advantage could be the combination of the two different pathological processes (amyloid and tau-pathology) in one parameter, thus simplifying the interpretation of results. Some studies report identical diagnostic performances for the heterologic Aβ_1-42_/pTau(181) and the amyloid Aβ_1-42_/Aβ_1-40_ ratio [34,35]. In other studies, the heterologic ratios Aβ_1-42_/pTau(181) [23,35,36] and Aβ_1-42_/tTau [23,36,37] slightly outperform the Aβ_1-42_/Aβ_1-40_ ratio. In this study, the AUC for pTau(181) determination yielded higher values than for any of the three biomarker ratios. This has also been previously shown for AUC values for pTau(181) and the Aβ_1-42_/Aβ_1-40_ ratio [23,37], while other studies found better values for the Aβ_1-42_/pTau(181) and Aβ_1-42_/tTau ratios than for pTau(181). Although heterologic biomarker ratios presumably show a slightly better performance than the Aβ_1-42_/Aβ_1-40_ ratio, their use is more prone to systematic errors caused by pre-analytic effects [38]. Accumulation of systematic errors may even lead to a misdiagnosis of patients. Most scientific studies do not represent real-world settings adequately as they are monocentric and use CSF samples from long-term storage, which are processed in batches. Thereby, systematical and individual-related factors and inter-laboratory differences might be neglected and results should be critically evaluated. In contrast to the Aβ_1-42_/Aβ_1-40_ ratio, use of heterologic ratios is currently not recommended by diagnostic guidelines.

### 4.1 Limitations

The cut-offs determined in this monocentric study were not additionally validated using an independent cohort, which could be performed in a future multicentric study. The derived cut-offs are however of high quality because they were established using a large panel of 439 patients suffering from different forms of dementias representing an authentic cohort for differential diagnosis of AD.

Although the EUROIMMUN ChLIAs and the Lumipulse G assays were processed on fully automated instruments, analytical variations occurring in the different laboratories might potentially have resulted in lower concordance of measurement results.

Generally, correct sample collection and storage are crucial for the reliability of the test results, and test results should always be interpreted together with those of further diagnostic analyses and based on the clinical picture of the patient.

### 4.2 Conclusion

The newly developed EUROIMMUN ChLIAs for fully automated determination of AD-related biomarkers show good analytical performance characteristics. High agreement rates of results obtained using EUROIMMUN ChLIAs with those obtained using Lumipulse G assays were found. Thus, the novel ChLIAs provide reliable tools for supporting the diagnosis of AD in CSF samples.

## Financial support

This research received no specific grant from any funding agency, commercial or non-profit sectors.

## Data availability statement

The data that support the findings of this study are available from the corresponding author upon reasonable request.

## CRediT authorship contribution statement

**Katharina Römpler:** Writing – review & editing, Validation, Investigation, Data curation. **Philipp Arendt:** Writing – review & editing, Formal analysis, Conceptualization. **Britta Brix:** Writing – review & editing, Resources. **Viola Borchardt-Lohölter:** Writing – original draft, Visualization, Formal analysis. **Anette Schulz:** Writing – original draft, Visualization, Formal analysis. **Mandy Busse:** Writing – review & editing. **Stefan Busse:** Writing – review & editing, Validation, Investigation.

## Declaration of competing interest

KR, PA, BB, VBL, and AS are or have been employees of EUROIMMUN Medizinische Labordiagnostika AG, Luebeck, Germany, a company that manufactures diagnostic tests and instruments. None of the authors benefits from any potential or actual financial or non-financial gain as a result of the work.

## Figures and Tables

**figs1:**
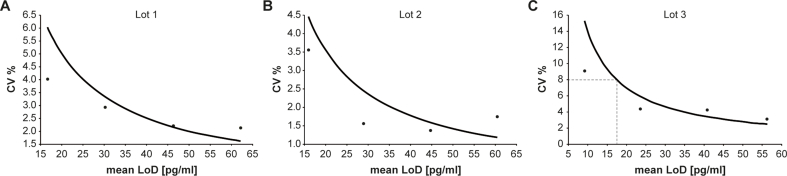


**figs2:**
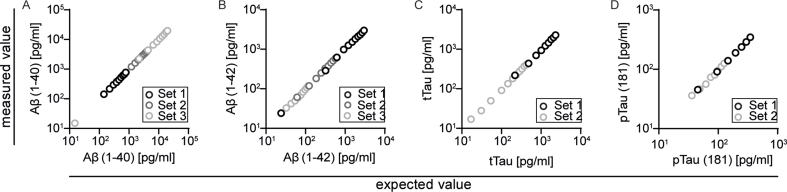


## Data Availability

Data will be made available on request.
