# Supplementary material for: Evaluation of the EUROIMMUN automated chemiluminescence immunoassays for measurement of four core biomarkers for Alzheimer’s disease in cerebrospinal fluid
Source: Pract Lab Med. 2024 Sep 5;41:e00425. doi: 10.1016/j.plabm.2024.e00425 (PMC11417521; doi:10.1016/j.plabm.2024.e00425)
Supplement: Multimedia component 1 [file mmc1.docx]

**Supplementary Figures**

**Supplementary Figure 1:** Plots showing LLoQ determination with LoD mean values plotted against respective CVs. LLoQ, which is equal to the concentration at the target limit of a CV of 8%, was undeterminable using lots 1 and 2.

**Supplementary Figure 2**: Representative graphs showing analysis of linearity using one lot of each Alzheimer ChLIAs for the determination of Aβ_1-40_ (181.7−19,685.4pg/ml), Aβ_1-42_ (32.6−2,628.6pg/ml), tTau (43.0−2,283.0pg/ml), and pTau(181) (53.0−337.2pg/ml) in different sets containing serially diluted samples.
