# Supplementary material for: Evaluation of the EUROIMMUN automated chemiluminescence immunoassays for measurement of four core biomarkers for Alzheimer’s disease in cerebrospinal fluid
Source: Pract Lab Med. 2024 Sep 5;41:e00425. doi: 10.1016/j.plabm.2024.e00425 (PMC11417521; doi:10.1016/j.plabm.2024.e00425)
Supplement: Multimedia component 2 [file mmc2.docx]

**Supplementary table 1:** Epitopes of AD-related biomarkers for binding of capture and detection Antibodies used in the respective ChLIAs. Ab, antibody

| **Analyte** | **Capture Ab epitope** | **Detection Ab epitope** |
| --- | --- | --- |
| **Aβ_1-40_** | 40 specific | 1 specific |
| **Aβ_1-42_** | 42 specific | 1 specific |
| **tTau** | Proline rich domain | N-terminal tau |
| **pTau(181)** | Phosphorylated Threonine 181 specific | N-terminal tau |
