# Supplementary material for: Evaluation of the EUROIMMUN automated chemiluminescence immunoassays for measurement of four core biomarkers for Alzheimer’s disease in cerebrospinal fluid
Source: Pract Lab Med. 2024 Sep 5;41:e00425. doi: 10.1016/j.plabm.2024.e00425 (PMC11417521; doi:10.1016/j.plabm.2024.e00425)
Supplement: Multimedia component 3 [file mmc3.docx]

**Supplementary table 2:** Intra-lot precision for AD-related biomarker ChLIAs including data on repeatability within a test run (within-run), reproducibility between test runs (between-run), reproducibility within a day (within-day) and on different days (between-day) and overall intra-lot precision. CV, coefficient of variation.

|  | **Sample** | **Mean (n=80)** | **Within-run** | **Between-run** | **Within-day** | **Between-day** | **Overall intra-lot precision** |
| --- | --- | --- | --- | --- | --- | --- | --- |
|  |  | **pg/ml** | **CV** | **CV** | **CV** | **CV** | **CV** |
| **Beta-Amyloid (1-40) ChLIA** | 1 | 2 594.2 | 2.4% | 1.1% | 2.6% | 1.9% | 3.2% |
|  | 2 | 3 821.2 | 2.5% | 0.2% | 2.5% | 1.3% | 2.8% |
|  | 3 | 7 700.3 | 2.8% | 2.0% | 3.4% | 0% | 3.4% |
|  | 4 | 9 594.0 | 2.3% | 2.3% | 3.3% | 0% | 3.3% |
|  | 5 | 13 013.2 | 1.4% | 1.8% | 2.2% | 0.8% | 2.4% |
|  | 6 | 26 547.3 | 6.8% | 0.9% | 6.9% | 4.0% | 8.0% |
| **Beta-Amyloid (1-42) ChLIA** | 1 | 273.8 | 2.0% | 0.8% | 2.2% | 1.1% | 2.4% |
|  | 2 | 619.9 | 2.3% | 0% | 2.3% | 1.4% | 2.7% |
|  | 3 | 781.7 | 2.8% | 1.6% | 3.2% | 2.1% | 3.8% |
|  | 4 | 985.9 | 2.2% | 1.6% | 2.7% | 0.7% | 2.8% |
|  | 5 | 1 289.7 | 1.8% | 0.5% | 1.8% | 1.0% | 2.1% |
|  | 6 | 2 343.9 | 4.2% | 0% | 4.2% | 2.0% | 4.7% |
| **Total-Tau ChLIA** | 1 | 137.8 | 2.0% | 1.2% | 2.3% | 1.9% | 3.0% |
|  | 2 | 215.8 | 1.4% | 1.1% | 1.7% | 1.7% | 2.4% |
|  | 3 | 462.3 | 1.0% | 1.2% | 1.6% | 1.1% | 1.9% |
|  | 4 | 948.3 | 1.4% | 0.4% | 1.5% | 1.2% | 1.9% |
|  | 5 | 1 223.0 | 1.2% | 1.5% | 1.9% | 1.2% | 2.3% |
|  | 6 | 1 798.5 | 2.0% | 1.7% | 2.6% | 2.0% | 3.3% |
| **pTau(181) ChLIA** | 1 | 26.1 | 2.6% | 3.4% | 4.3% | 2.5% | 5.0% |
|  | 2 | 41.2 | 2.0% | 1.5% | 2.5% | 2.4% | 3.5% |
|  | 3 | 48.8 | 1.7% | 1.5% | 2.3% | 2.8% | 3.6% |
|  | 4 | 74.2 | 1.3% | 0.5% | 1.4% | 2.4% | 2.8% |
|  | 5 | 203.6 | 1.3% | 1.4% | 1.9% | 2.2% | 2.9% |
|  | 6 | 323.6 | 1.4% | 0.3% | 1.4% | 2.1% | 2.5% |
