# Supplementary material for: Evaluation of the EUROIMMUN automated chemiluminescence immunoassays for measurement of four core biomarkers for Alzheimer’s disease in cerebrospinal fluid
Source: Pract Lab Med. 2024 Sep 5;41:e00425. doi: 10.1016/j.plabm.2024.e00425 (PMC11417521; doi:10.1016/j.plabm.2024.e00425)
Supplement: Multimedia component 4 [file mmc4.docx]

**Supplementary table 3**: Inter-lot precision for AD-related biomarker ChLIAs with data on reproducibility using different lots (between-lot) and overall inter-lot precision. CV, coefficient of variation.

|  | **Sample** | **Mean (n=90)** | **Between-lot** | **Overall inter-lot precision** |
| --- | --- | --- | --- | --- |
|  |  | **pg/ml** | **CV** | **CV** |
| **Beta-Amyloid (1-40) ChLIA** | 1 | 2702.0 | 2.6% | 4.1% |
|  | 2 | 3954.3 | 3.2% | 4.2% |
|  | 3 | 7840.3 | 3.0% | 4.9% |
|  | 4 | 9758.9 | 1.8% | 3.6% |
|  | 5 | 12785.6 | 1.7% | 4.2% |
|  | 6 | 23022.3 | 13.1% | 14.0% |
| **Beta-Amyloid (1-42) ChLIA** | 1 | 282.9 | 4.2% | 5.4% |
|  | 2 | 640.3 | 3.8% | 4.9% |
|  | 3 | 804.6 | 4.6% | 5.8% |
|  | 4 | 1018.8 | 4.4% | 5.4% |
|  | 5 | 1323.6 | 3.0% | 4.8% |
|  | 6 | 2449.9 | 4.6% | 6.0% |
| **Total-Tau ChLIA** | 1 | 136.9 | 2.7% | 3.9% |
|  | 2 | 216.1 | 1.8% | 3.6% |
|  | 3 | 473.3 | 3.6% | 4.9% |
|  | 4 | 978.5 | 3.8% | 5.0% |
|  | 5 | 1228.1 | 1.9% | 3.8% |
|  | 6 | 1823.5 | 3.4% | 5.0% |
| **pTau(181) ChLIA** | 1 | 27.7 | 7.3% | 8.3% |
|  | 2 | 41.9 | 4.8% | 5.9% |
|  | 3 | 50.1 | 4.0% | 5.2% |
|  | 4 | 75.0 | 4.1% | 4.9% |
|  | 5 | 206.1 | 4.4% | 5.2% |
|  | 6 | 326.9 | 4.5% | 5.3% |
