# Supplementary material for: Evaluation of the EUROIMMUN automated chemiluminescence immunoassays for measurement of four core biomarkers for Alzheimer’s disease in cerebrospinal fluid
Source: Pract Lab Med. 2024 Sep 5;41:e00425. doi: 10.1016/j.plabm.2024.e00425 (PMC11417521; doi:10.1016/j.plabm.2024.e00425)
Supplement: Multimedia component 5 [file mmc5.docx]

**Supplementary table 4**: Range of detectability LoB, LoD and LoQ and measurement range for the four EUROIMMUN ChLIA systems; n.d., non-determinable.

|  | **Lot** | **LoB [pg/ml]** | **LoD [pg/ml]** | **LoQ [pg/ml]** | **Measurement range [pg/ml]** |
| --- | --- | --- | --- | --- | --- |
| **Beta-Amyloid (1-40) ChLIA** | 1 | **15.4** | **28.3** | **41.0** | 41.0 – 20,000 |
|  | 2 | 8.5 | 19.0 | 19.0 |  |
|  | 3 | 8.9 | 13.9 | 14.1 |  |
| **Beta-Amyloid (1-42) ChLIA** | 1 | 12.1 | 16.9 | 36.7 | 45.4 – 2,000 |
|  | 2 | **12.7** | **17.5** | **45.4** |  |
|  | 3 | 11.8 | 16.2 | 31.4 |  |
| **Total-Tau ChLIA** | 1 | **3.9** | **5.6** | n.d. | 17.4 – 2,000 |
|  | 2 | 0.0 | 1.2 | n.d. |  |
|  | 3 | 2.6 | 4.9 | **17.4** |  |
| **pTau(181) ChLIA** | 1 | 0.0 | 0.9 | 4.7 | 9.2 – 400 |
|  | 2 | **1.8** | **3.1** | **9.2** |  |
|  | 3 | 0.4 | 1.3 | 5.7 |  |
