# Supplementary material for: Evaluation of the EUROIMMUN automated chemiluminescence immunoassays for measurement of four core biomarkers for Alzheimer’s disease in cerebrospinal fluid
Source: Pract Lab Med. 2024 Sep 5;41:e00425. doi: 10.1016/j.plabm.2024.e00425 (PMC11417521; doi:10.1016/j.plabm.2024.e00425)
Supplement: Multimedia component 6 [file mmc6.docx]

**Supplementary table 5:** Interferences of blood (1%) and biotin (up to 10µg/ml) in samples tested with the four EUROIMMUN ChLIAs.

|  | **Blood** | | **Biotin** | |
| --- | --- | --- | --- | --- |
|  | **Recovery (range, %)** | **Recovery (mean, %)** | **Recovery (range, %)** | **Recovery (mean, %)** |
| **Beta-Amyloid (1-40) ChLIA** | 98.5 - 104.4 | 101.1 | 96.0 - 102.9 | 99.8 |
| **Beta-Amyloid (1-42) ChLIA** | 96.1 - 103.3 | 99.0 | 83.6 - 103.1 | 95.7 |
| **Total-Tau ChLIA** | 89.9 - 101.5 | 95.4 | 92.6 - 107.1 | 99.4 |
| **pTau(181) ChLIA** | 83.9 - 98.4 | 90.1 | 97.0 - 103.0 | 99.9 |
