# Supplementary material for: Evaluation of the EUROIMMUN automated chemiluminescence immunoassays for measurement of four core biomarkers for Alzheimer’s disease in cerebrospinal fluid
Source: Pract Lab Med. 2024 Sep 5;41:e00425. doi: 10.1016/j.plabm.2024.e00425 (PMC11417521; doi:10.1016/j.plabm.2024.e00425)
Supplement: Multimedia component 7 [file mmc7.docx]

**Supplementary table 6:** Results for stability testing of EUROIMMUN ChLIAs.

| **Stability** | **Beta-Amyloid**  **(1-40) ChLIA** | **Beta-Amyloid**  **(1-42) ChLIA** | **Total-Tau ChLIA** | **pTau(181) ChLIA** |
| --- | --- | --- | --- | --- |
| **Transport stability** | 2°C – 33°C | 2°C – 33°C | 2°C – 33°C | 2°C – 33°C |
| **Accelerated stability** | 12 days | 12 days | 11 days | 11 days |
| **Calibration stability** | 28 days | 28 days | 28 days | 28 days |
| **On-board stability**  **cartridges** | 60 days | 60 days | 60 days | 60 days |
| **On-board stability**  **calibrators** | 6 hours | 6 hours | 10 hours | 10 hours |
| **In-use stability**  **calibrators** | 60 days | 60 days | 60 days | 60 days |
| **Freeze/thaw cycles**  **calibrators** | 3 cycles | 3 cycles | 5 cycles | 5 cycles |
| **Real-time stability in original packing (Cartridges and calibrators)** | 24 months | 24 months | 24 months* | 24 months |

* Shown for 2/3 lots up to this date, 3^rd^ analysis ongoing
