# Supplementary material for: Evaluation of the EUROIMMUN automated chemiluminescence immunoassays for measurement of four core biomarkers for Alzheimer’s disease in cerebrospinal fluid
Source: Pract Lab Med. 2024 Sep 5;41:e00425. doi: 10.1016/j.plabm.2024.e00425 (PMC11417521; doi:10.1016/j.plabm.2024.e00425)
Supplement: Multimedia component 8 [file mmc8.docx]

**Supplementary table 7**: Trueness between replicate quantitative measurements and reference quantitative values obtained with the Beta-Amyloid (1-42) ChLIA. The mean value is the average of three replicate measurements obtained using three lots.

|  | **CRM** | **Reference concentration [pg/ml]** | **Lot** | **Mean concentration**  **[pg/ml]** | **Recovery**  **[%]** |
| --- | --- | --- | --- | --- | --- |
| **Beta-Amyloid (1-42) ChLIA** | 1 | 450.0 | 1 | 496.9 | 110.4 |
|  |  |  | 2 | 444.3 | 98.7 |
|  |  |  | 3 | 453.6 | 100.8 |
|  | 2 | 720.0 | 1 | 754.7 | 104.8 |
|  |  |  | 2 | 711.7 | 98.8 |
|  |  |  | 3 | 755.7 | 105.0 |
|  | 3 | 1220.0 | 1 | 1200.4 | 98.4 |
|  |  |  | 2 | 1112.5 | 91.2 |
|  |  |  | 3 | 1130.8 | 92.7 |
