# Supplementary material for: Evaluation of the EUROIMMUN automated chemiluminescence immunoassays for measurement of four core biomarkers for Alzheimer’s disease in cerebrospinal fluid
Source: Pract Lab Med. 2024 Sep 5;41:e00425. doi: 10.1016/j.plabm.2024.e00425 (PMC11417521; doi:10.1016/j.plabm.2024.e00425)
Supplement: Multimedia component 9 [file mmc9.docx]

**Supplementary table 8:** Results of cross-reactivity analysis for Beta-Amyloid (1-40), Beta-Amyloid (1-42) and pTau(181) ChLIAs.

| **Substance**  **[10,000 pg/ml]** | **Recovery rate of structurally related antigen [%]** | |
| --- | --- | --- |
|  | **Beta-Amyloid (1-40) ChLIA** | **Beta-Amyloid (1-42) ChLIA** |
| Aβ_1-38_ | <0.1 | <0.1 |
| Aβ_1-39_ | 11.0 | <0.1 |
| Aβ_1-40_ | − | <0.1 |
| Aβ_1-42_ | <0.1 | − |
| Aβ_2-42_ | <0.1 | 0.6 |
| Aβ_3-42_ | 0.4 | <0.1 |
| Aβ_4-42_ | <0.1 | 0.3 |
| Aβ_11-42_ | <0.1 | <0.1 |
| Aβ_1-43_ | 0.1 | 0.3 |
| **Amount of Tau441 [pg/ml]** | **pTau(181) ChLIA** | |
| 200 | <0.02 | |
| 2000 | <0.02 | |
| 20000 | <0.02 | |
