# Supplementary material for: Evaluation of the EUROIMMUN automated chemiluminescence immunoassays for measurement of four core biomarkers for Alzheimer’s disease in cerebrospinal fluid
Source: Pract Lab Med. 2024 Sep 5;41:e00425. doi: 10.1016/j.plabm.2024.e00425 (PMC11417521; doi:10.1016/j.plabm.2024.e00425)
Supplement: Multimedia component 10 [file mmc10.docx]

**Supplementary Table 9**: Cut-off determination for (A) the Beta-Amyloid (1-42), tTau and pTau(181) ChLIAs and (B) for Aβ_1-42_/Aβ_1-40_, Aβ_1-42_/tTau, and Aβ_1-42_/pTau(181) ratios. Concentration (A) of the analyte and ratio (B) determined using the respective ChLIAs, sensitivity, specificity, and Youden’s indices are listed. Bold entries indicate the most balanced ratio of sensitivity to specificity with maximum Youden’s index.

A)

|  | **Concentration [pg/ml]** | **Sensitivity**  **[%]** | **Specificity**  **[%]** | **Youden’s index** |
| --- | --- | --- | --- | --- |
| **Beta-Amyloid (1-42) ChLIA** | 1802 | 100.0 | 0.5 | 0.005 |
|  | 740 | 84.0 | 62.7 | 0.467 |
|  | **741** | **84.5** | **62.7** | **0.472** |
|  | 741 | 84.5 | 62.3 | 0.467 |
|  | 744 | 84.5 | 61.8 | 0.463 |
|  | 744 | 84.9 | 61.8 | 0.467 |
|  | 749 | 84.9 | 61.4 | 0.463 |
|  | 752 | 85.4 | 61.4 | 0.468 |
|  | 221 | 1.4 | 100.0 | 0.014 |
| **Total-Tau ChLIA** | 215 | 100.0 | 19.5 | 0.195 |
|  | 434 | 92.2 | 74.1 | 0.663 |
|  | 479 | 87.2 | 80.9 | 0.681 |
|  | 489 | 86.3 | 81.8 | 0.681 |
|  | 499 | 84.9 | 83.2 | 0.681 |
|  | **508** | **83.6** | **84.5** | **0.681** |
|  | 555 | 79.9 | 88.2 | 0.681 |
|  | 593 | 74.4 | 90.9 | 0.653 |
|  | 3709 | 0.0 | 99.5 | -0.005 |
| **pTau(181) ChLIA** | 21.3 | 100.0 | 20.5 | 0.205 |
|  | 48.9 | 95.4 | 82.3 | 0.777 |
|  | 54.4 | 93.2 | 86.4 | 0.795 |
|  | 56.3 | 92.2 | 87.3 | 0.795 |
|  | 57.1 | 91.8 | 87.7 | 0.795 |
|  | 57.4 | 91.3 | 88.2 | 0.795 |
|  | **58.2** | **90.9** | **88.6** | **0.795** |
|  | 74.6 | 79.0 | 92.7 | 0.717 |
|  | 194.2 | 14.6 | 100.0 | 0.146 |

B)

|  | **Ratio** | **Sensitivity**  **[%]** | **Specificity**  **[%]** | **Youden’s index** |
| --- | --- | --- | --- | --- |
| **Aβ_1-42_/Aβ_1-40_** | 0.162 | 100.0 | 9.5 | 0.095 |
|  | 0.092 | 85.8 | 86.4 | 0.722 |
|  | 0.092 | 86.3 | 86.4 | 0.727 |
|  | **0.093** | **86.8** | **86.4** | **0.731** |
|  | 0.093 | 86.8 | 85.9 | 0.727 |
|  | 0.093 | 86.8 | 85.5 | 0.722 |
|  | 0.058 | 21.5 | 100.0 | 0.215 |
| **Aβ_1-42_/tTau** | 0.04 | 0.000 | 1.000 | 0.000 |
|  | 0.79 | 0.626 | 0.950 | 0.576 |
|  | 1.17 | 0.890 | 0.882 | 0.772 |
|  | 1.19 | 0.895 | 0.877 | 0.772 |
|  | 1.21 | 0.900 | 0.873 | 0.772 |
|  | 1.24 | 0.904 | 0.868 | 0.772 |
|  | 1.27 | 0.909 | 0.864 | 0.772 |
|  | **1.28** | **0.913** | **0.864** | **0.777** |
|  | 1.29 | 0.913 | 0.859 | 0.772 |
|  | 1.35 | 0.922 | 0.850 | 0.772 |
|  | 1.64 | 0.950 | 0.777 | 0.727 |
|  | 4.39 | 1.000 | 0.109 | 0.109 |
| **Aβ_1-42_/tTau** | 2.67 | 0.192 | 1.000 | 0.192 |
|  | 6.38 | 0.753 | 0.950 | 0.703 |
|  | 8.95 | 0.872 | 0.895 | 0.768 |
|  | 8.95 | 0.877 | 0.891 | 0.768 |
|  | 9.06 | 0.881 | 0.891 | 0.772 |
|  | 9.30 | 0.881 | 0.886 | 0.768 |
|  | **9.50** | **0.886** | **0.886** | **0.772** |
|  | 10.01 | 0.886 | 0.882 | 0.768 |
|  | 10.04 | 0.890 | 0.882 | 0.772 |
|  | 10.06 | 0.890 | 0.877 | 0.768 |
|  | 10.85 | 0.900 | 0.868 | 0.768 |
|  | 10.98 | 0.904 | 0.868 | 0.772 |
|  | 11.28 | 0.904 | 0.864 | 0.768 |
|  | 11.94 | 0.913 | 0.855 | 0.768 |
|  | 12.43 | 0.918 | 0.850 | 0.768 |
|  | 12.79 | 0.927 | 0.841 | 0.768 |
|  | 19.89 | 0.950 | 0.723 | 0.672 |
|  | 45.41 | 1.000 | 0.145 | 0.145 |
